# Supplementary figures and images for: A comparison of diceCT and histology for determination of nasal epithelial type
Source: PeerJ. 2021 Nov 3;9:e12261. doi: 10.7717/peerj.12261 (PMC8571959; doi:10.7717/peerj.12261)

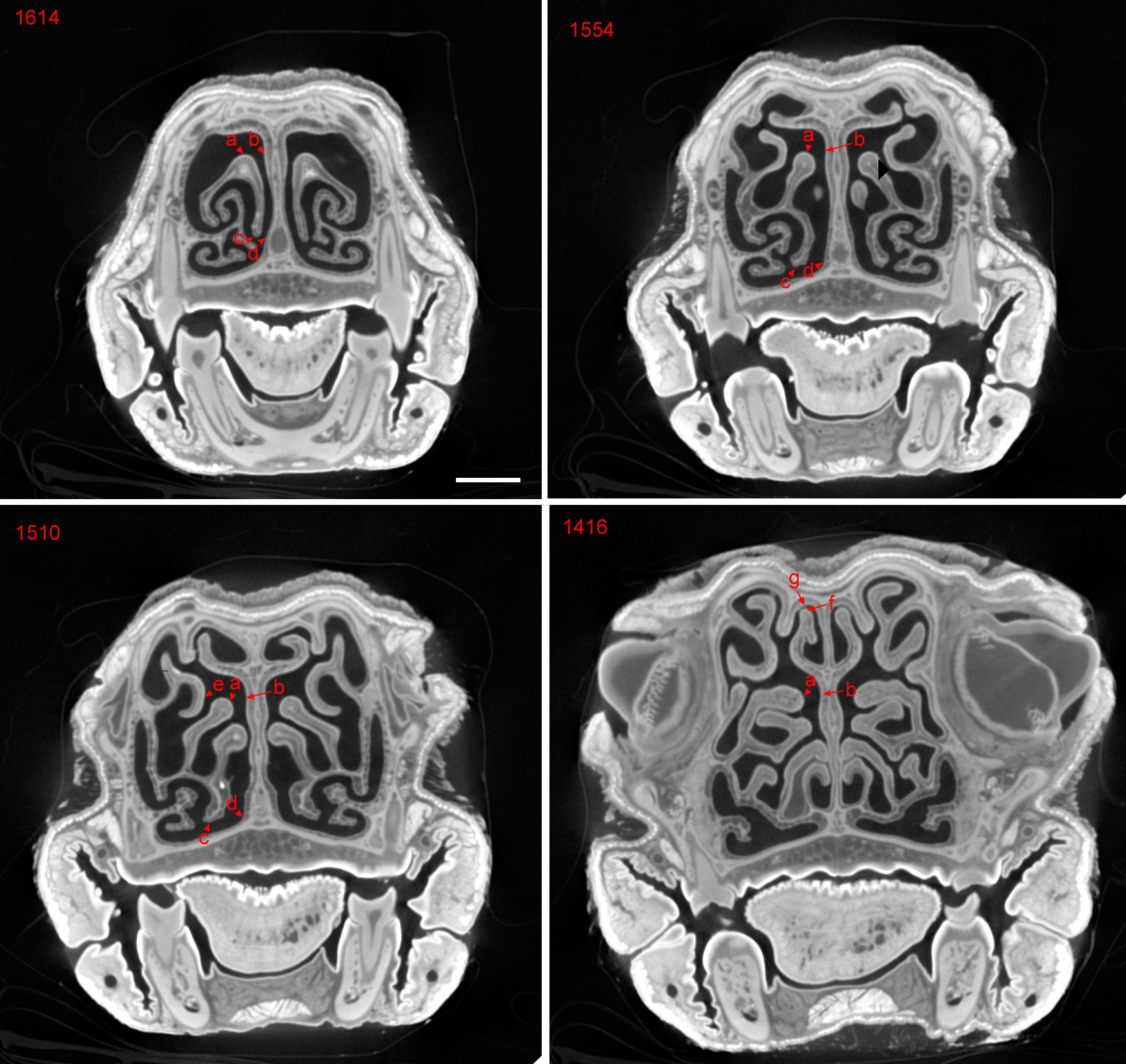

Supplement: Supplemental Information 1 — DiceCT slices of Cynopterus sphinx, showing sites measured for epithelial thickness (numbers indicate slice numbers; higher numbers are more rostral). Measurements were made on histological sections in the same region as this slice series. Site a: ridge or apex of ET I, which orients superiorly at rostral extent (1,614) and then leans medially as ET I is followed caudally. Site b: septal epithelium found adjacent to site a. Site c,inferior-most limit of medial lamina of ET I. Site d: septal epithelium found adjacent to site d. Site e, medial side of the frontoburbinal. Site f: superior-most tip of the nasoturbinal. Site g: “roof” of nasal cavity, opposite site f. Locations c and d were selected as ventral sites where respiratory epithelium is expected. [file peerj-09-12261-s001.png]

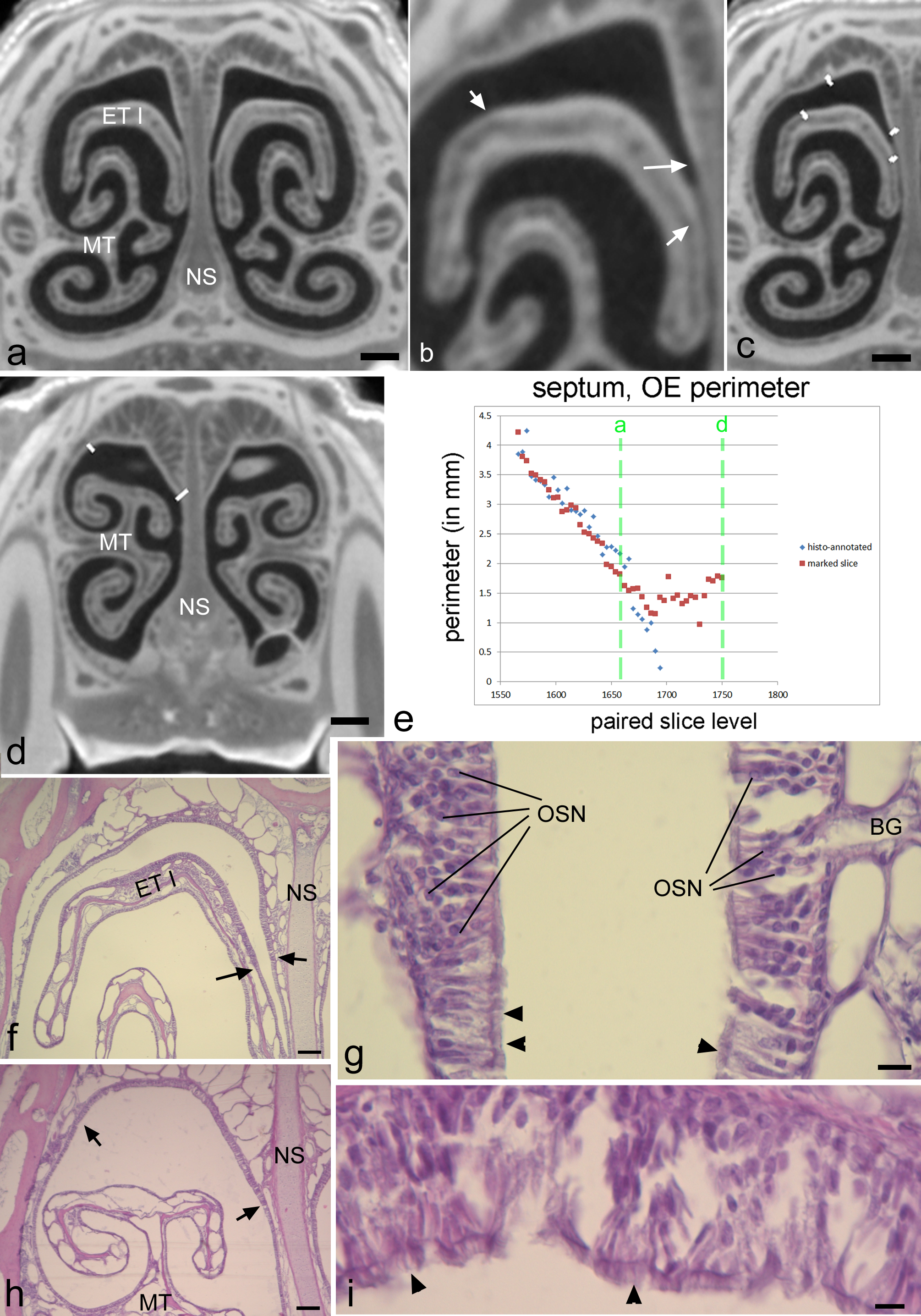

Supplement: Supplemental Information 2 — DiceCT (A–D) and histological views (F–I) of the nasal cavity in adult Cynopterus. After aligning CT to histology, the diceCT slices were assessed for changes in nasal epithelial thickness. (A) In the region of the rostral projection of the first ethmoturbinal (ET I), noticeably thicker epithelium is observed on the superior side of ET I, and on adjacent surfaces of the nasal septum (NS) and “roof” of the nasal cavity (b, arrows indicate transitions). An observer who was blind to the histology marked where the change in thickness was evident using ImageJ software (C). (D) Rostral to ET I, thicker epithelium exists on the roof of the nasal cavity (borders annotated). (E) approximate levels of slices in plate a and d are indicated by green dashed lines on the graph of septal OE. (F, G) Low and higher magnification views of the histology section matching plate a. Note a transition in epithelial thickness can be detected at low magnification (arrows). The thicker epithelium has numerous rows of nuclei of olfactory sensory neurons (OSN), and the transition to thinner non-OE is nearly abrupt, with a shift to respiratory epithelium (arrowheads indicate cilia). (H, I) Low and higher magnification of histology matching the slice in plate d. Note a change in thickness can be seen at low magnification. However, this epithelium is ciliated (arrowheads) indicating it is a respiratory type. BG, Bowman’s glands; MT, maxilloturbinal. Scale bars, a–d, 0.5 mm; f, h, 150 µm; g, I, 10 µm. [file peerj-09-12261-s002.png]

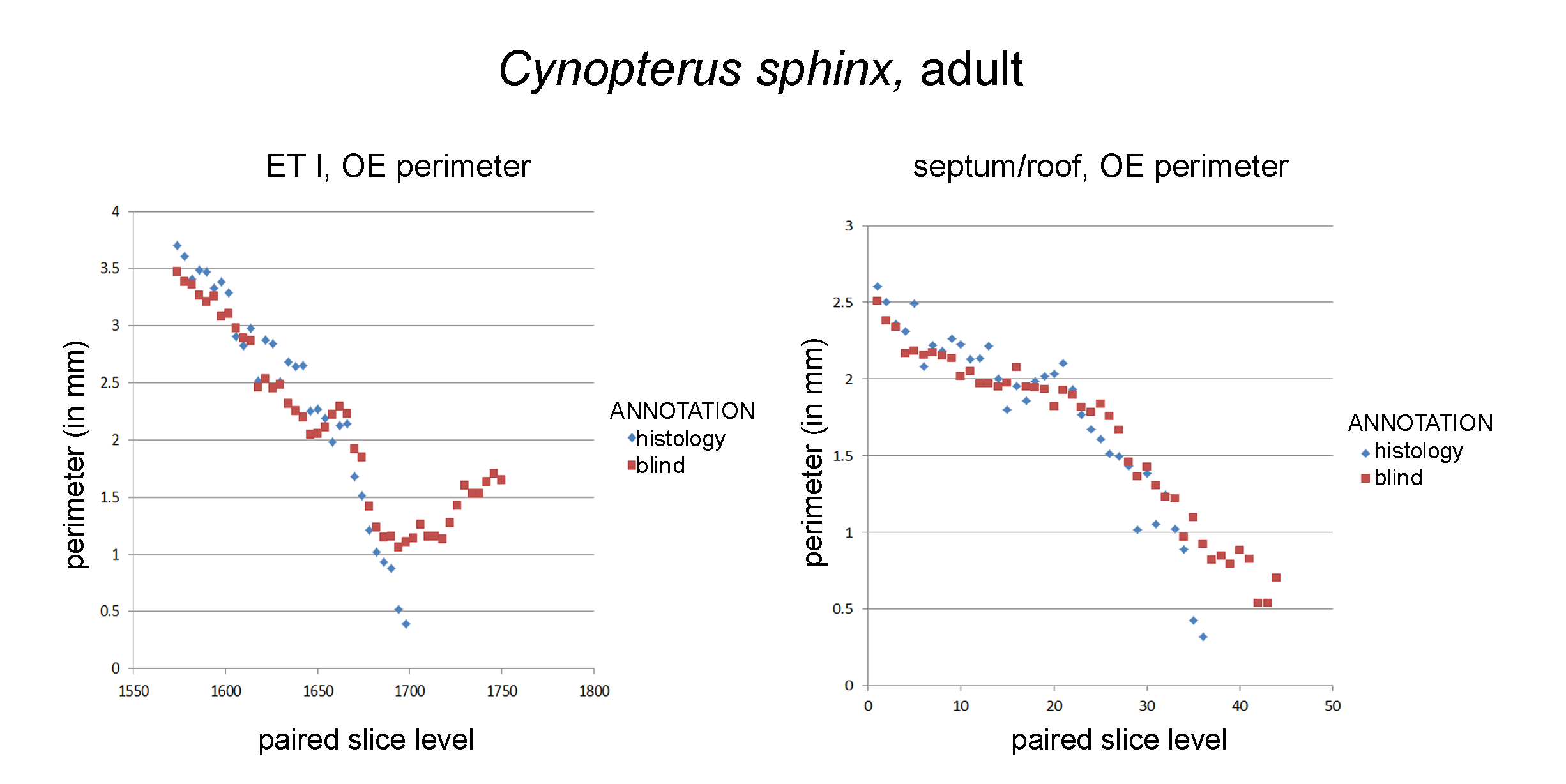

Supplement: Supplemental Information 3 — Olfactory epithelial (OE) perimeter on the first ethmoturbinals (ET I) and the nasal septum as measured based on diceCT slices annotated blindly, based on epithelial thickness (“blind”) and diceCT annotated based on histology (“histology”). (A) OE as assessed by the two methods was close for most of the length of ET I, but the methods diverged anteriorly (right side of the graph). A similar phenomena was observed for septal measurements (B). [file peerj-09-12261-s003.png]
