# Supplementary material for: A comparison of diceCT and histology for determination of nasal epithelial type
Source: PeerJ. 2021 Nov 3;9:e12261. doi: 10.7717/peerj.12261 (PMC8571959; doi:10.7717/peerj.12261)
Supplement: Supplemental Information 4 [file peerj-09-12261-s004.docx]

| **Table S1: CT-based versus histology-based measurements of maxilloturbinal perimeter** | | | | | |
| --- | --- | --- | --- | --- | --- |
| matching  levels | Perimeter (in mm) | | | Difference:  µCT - diceCT | Difference:  µCT – histo. |
|  | µCT | diceCT | Histological* |  |  |
| 1 | 3.31 | 3.333 |  | -0.023 |  |
| 2 | 3.197 | 3.283 | 2.945 | -0.086 | 0.252 |
| 3 | 3.163 | 3.232 | 3.015 | -0.069 | 0.148 |
| 4 | 3.277 | 3.192 |  | 0.085 |  |
| 5 | 3.194 | 3.124 | 2.872 | 0.07 | 0.322 |
| 6 | 3.262 | 2.97 |  | 0.292 |  |
| 7 | 3.111 | 2.92 | 2.875 | 0.191 | 0.236 |
| 8 | 3.141 | 2.863 |  | 0.278 |  |
| 9 | 3.075 | 2.847 | 2.664 | 0.228 | 0.411 |
| 10 | 2.975 | 2.896 |  | 0.079 |  |
| 11 | 2.942 | 2.796 | 2.69 | 0.146 | 0.252 |
| 12 | 2.795 | 2.716 |  | 0.079 |  |
| 13 | 2.891 | 2.687 | 2.571 | 0.204 | 0.32 |
| 14 | 2.783 | 2.664 |  | 0.119 |  |
| 15 | 2.714 | 2.627 | 2.43 | 0.087 | 0.284 |
| 16 | 2.73 | 2.585 |  | 0.145 |  |
| 17 | 2.713 | 2.569 | 2.395 | 0.144 | 0.318 |
| 18 | 2.667 | 2.58 |  | 0.087 |  |
| 19 | 2.637 | 2.528 | 2.285 | 0.109 | 0.352 |
| 20 | 2.59 | 2.548 |  | 0.042 |  |
| 21 | 2.556 | 2.447 | 2.273 | 0.109 | 0.283 |
| 22 | 2.569 | 2.438 |  | 0.131 |  |
| 23 | 2.554 | 2.466 | 2.18 | 0.088 | 0.374 |
| 24 | 2.526 | 2.437 |  | 0.089 |  |
| 25 | 2.373 | 2.337 | 2.105 | 0.036 | 0.268 |
| 26 | 2.419 | 2.321 |  | 0.098 |  |
| 27 | 2.399 | 2.334 | 2.101 | 0.065 | 0.298 |
| 28 | 2.38 | 2.213 |  | 0.167 |  |
| 29 | 2.31 | 2.252 | 2.071 | 0.058 | 0.239 |
| 30 | 2.36 | 2.222 |  | 0.138 |  |
| 31 | 2.343 | 2.221 | 1.933 | 0.122 | 0.41 |
| 32 | 2.28 | 2.26 |  | 0.02 |  |
| 33 | 2.224 | 2.108 | 1.968 | 0.116 | 0.256 |
| 34 | 2.224 | 2.104 |  | 0.12 |  |
| 35 | 2.161 | 2.095 | 1.861 | 0.066 | 0.3 |
| 36 | 2.36 | 2.049 |  | 0.311 |  |
| 37 | 2.19 | 1.996 | 1.773 | 0.194 | 0.417 |
| 38 | 2.118 | 1.949 |  | 0.169 |  |
| 39 | 2.059 | 1.960 | 1.742 | 0.099 | 0.317 |
| 40 | 2.009 | 1.896 |  | 0.113 |  |
| 41 | 1.989 | 1.849 | 1.681 | 0.14 | 0.308 |
| 42 | 1.974 | 1.846 |  | 0.128 |  |
| 43 | 1.997 | 1.886 | 1.535 | 0.111 | 0.462 |
| 44 | 1.904 | 1.785 |  | 0.119 |  |
| 45 | 1.918 | 1.81 | 1.529 | 0.108 | 0.389 |
| 46 | 1.894 | 1.805 |  | 0.089 |  |
| 47 | 1.819 | 1.786 | 1.507 | 0.033 | 0.312 |
| 48 | 1.856 | 1.705 |  | 0.151 |  |
| 49 | 1.812 | 1.646 | 1.495 | 0.166 | 0.317 |
| 50 | 1.788 | 1.599 |  | 0.189 |  |
| 51 | 1.704 | 1.661 | 1.442 | 0.043 | 0.262 |
| 52 | 1.728 | 1.616 |  | 0.112 |  |
| 53 | 1.73 | 1.593 | 1.262 | 0.137 | 0.468 |
|  |  |  | average | 0.125204 | 0.327 |

*, only selected histological sections matched the CT slice levels.
