# Supplementary material for: A comparison of diceCT and histology for determination of nasal epithelial type
Source: PeerJ. 2021 Nov 3;9:e12261. doi: 10.7717/peerj.12261 (PMC8571959; doi:10.7717/peerj.12261)
Supplement: Supplemental Information 5 [file peerj-09-12261-s005.docx]

| **Table S2: CT-based versus histology-based measurements of epiturbinal perimeter** | | | | | |
| --- | --- | --- | --- | --- | --- |
|  | perimeter (mm) | | |  |  |
| matching levels | µCT | diceCT | histology | Difference:  µCT - diceCT | Difference:  µCT – histo. |
| 1 |  |  |  |  |  |
| 2 |  |  |  |  |  |
| 3 | 0.767 | 0.633 |  | 0.134 |  |
| 4 | 1.184 | 1.085 | 0.861 | 0.099 | 0.323 |
| 5 | 1.541 | 1.419 |  | 0.122 |  |
| 6 | 1.801 | 1.697 | 1.58 | 0.104 | 0.221 |
| 7 | 1.964 | 1.92 |  | 0.044 |  |
| 8 | 2.215 | 2.109 |  | 0.106 |  |
| 9 | 2.376 | 2.277 | 2.07 | 0.099 | 0.306 |
| 10 | 2.51 | 2.439 |  | 0.071 |  |
| 11 | 2.699 | 2.6 | 2.45 | 0.099 | 0.249 |
| 12 | 2.844 | 2.717 |  | 0.127 |  |
| 13 | 2.984 | 2.89 |  | 0.094 |  |
| 14 | 3.123 | 2.996 | 2.75 | 0.127 | 0.373 |
| 15 | 3.261 | 3.134 |  | 0.127 |  |
| 16 | 3.334 | 3.24 |  | 0.094 |  |
| 17 | 3.489 | 3.373 | 3.09 | 0.116 | 0.399 |
| 18 | 3.606 | 3.523 |  | 0.083 |  |
| 19 | 3.728 | 3.606 | 3.31 | 0.122 | 0.418 |
| 20 | 3.807 | 3.756 |  | 0.051 |  |
| 21 | 3.968 | 3.873 | 3.66 | 0.095 | 0.308 |
| 22 | 4.122 | 3.991 |  | 0.131 |  |
| 23 | 4.261 | 4.145 |  | 0.116 |  |
| 24 | 4.378 | 4.251 |  | 0.127 |  |
| 25 | 4.541 | 4.389 |  | 0.152 |  |
| 26 | 4.7 | 4.532 |  | 0.168 |  |
| Average difference | | | | 0.106 | 0.325 |

*, only selected histological sections matched the CT slice levels.
