# Supplementary material for: A comparison of diceCT and histology for determination of nasal epithelial type
Source: PeerJ. 2021 Nov 3;9:e12261. doi: 10.7717/peerj.12261 (PMC8571959; doi:10.7717/peerj.12261)
Supplement: Supplemental Information 6 [file peerj-09-12261-s006.docx]

| Table S3: Comparison of olfactory epithelial perimeter in *Cynopterus* using two methods | | | | | | |
| --- | --- | --- | --- | --- | --- | --- |
| Perimeter (mm) of OE on roof/septum roof/septum | | | | Perimeter (mm) of OE on ET I | | |
|  | Annotation | |  | Annotation | |  |
| Matching  levels | Histology^1^ | Blind^2^ | difference | histology | Blind | difference |
| 1 | 3.7 | 3.472 | 0.228 | 2.605 | 2.508 | 0.097 |
| 2 | 3.604 | 3.381 | 0.223 | 2.504 | 2.38 | 0.124 |
| 3 | 3.409 | 3.357 | 0.052 | 2.36 | 2.338 | 0.022 |
| 4 | 3.49 | 3.263 | 0.227 | 2.313 | 2.165 | 0.148 |
| 5 | 3.471 | 3.211 | 0.26 | 2.49 | 2.184 | 0.306 |
| 6 | 3.328 | 3.256 | 0.072 | 2.081 | 2.154 | -0.073 |
| 7 | 3.384 | 3.084 | 0.3 | 2.22 | 2.17 | 0.05 |
| 8 | 3.288 | 3.102 | 0.186 | 2.184 | 2.151 | 0.033 |
| 9 | 2.906 | 2.979 | -0.073 | 2.264 | 2.134 | 0.13 |
| 10 | 2.825 | 2.889 | -0.064 | 2.223 | 2.016 | 0.207 |
| 11 | 2.979 | 2.869 | 0.11 | 2.129 | 2.05 | 0.079 |
| 12 | 2.513 | 2.463 | 0.05 | 2.135 | 1.968 | 0.167 |
| 13 | 2.872 | 2.531 | 0.341 | 2.215 | 1.971 | 0.244 |
| 14 | 2.846 | 2.451 | 0.395 | 2.004 | 1.949 | 0.055 |
| 15 | 2.505 | 2.484 | 0.021 | 1.801 | 1.974 | -0.173 |
| 16 | 2.687 | 2.315 | 0.372 | 1.955 | 2.078 | -0.123 |
| 17 | 2.641 | 2.252 | 0.389 | 1.859 | 1.949 | -0.09 |
| 18 | 2.655 | 2.199 | 0.456 | 1.985 | 1.941 | 0.044 |
| 19 | 2.252 | 2.044 | 0.208 | 2.017 | 1.932 | 0.085 |
| 20 | 2.273 | 2.053 | 0.22 | 2.036 | 1.818 | 0.218 |
| 21 | 2.192 | 2.11 | 0.082 | 2.102 | 1.929 | 0.173 |
| 22 | 1.985 | 2.221 | -0.236 | 1.931 | 1.893 | 0.038 |
| 23 | 2.125 | 2.293 | -0.168 | 1.766 | 1.813 | -0.047 |
| 24 | 2.144 | 2.229 | -0.085 | 1.671 | 1.781 | -0.11 |
| 25 | 1.677 | 1.923 | -0.246 | 1.607 | 1.834 | -0.227 |
| 26 | 1.513 | 1.849 | -0.336 | 1.512 | 1.759 | -0.247 |
| 27 | 1.21 | 1.419 | -0.209 | 1.497 | 1.665 | -0.168 |
| 28 | 1.018 | 1.233 | -0.215 | 1.434 | 1.459 | -0.025 |
| 29 | 0.93 | 1.149 | -0.219 | 1.015 | 1.363 | -0.348 |
| 30 | 0.879 | 1.157 | -0.278 | 1.382 | 1.428 | -0.046 |
| 31 | 0.518 | 1.063 | -0.545 | 1.053 | 1.302 | -0.249 |
| 32 | 0.395 | 1.11 | -0.715 | 1.243 | 1.231 | 0.012 |
| 33 |  | 1.143 | -1.143 | 1.02 | 1.22 | -0.2 |
| 34 |  | 1.257 | -1.257 | 0.887 | 0.968 | -0.081 |
| 35 |  | 1.159 | -1.159 | 0.422 | 1.096 | -0.674 |
| 36 |  | 1.157 | -1.157 | 0.32 | 0.921 | -0.601 |
| 37 |  | 1.135 | -1.135 |  | 0.818 | -0.818 |
| 38 |  | 1.275 | -1.275 |  | 0.847 | -0.847 |
| 39 |  | 1.423 | -1.423 |  | 0.791 | -0.791 |
| 40 |  | 1.603 | -1.603 |  | 0.881 | -0.881 |
| 41 |  | 1.532 | -1.532 |  | 0.824 | -0.824 |
| 42 |  | 1.531 | -1.531 |  | 0.534 | -0.534 |
| 43 |  | 1.633 | -1.633 |  | 0.537 | -0.537 |
| 44 |  | 1.708 | -1.708 |  | 0.7 | -0.7 |
| 45 |  | 1.647 | -1.647 |  |  |  |
|  |  |  |  |  |  |  |
|  |  |  |  |  |  |  |
| Avg |  |  | -0.524 |  | avg | -0.163 |
| 1, Based on annotations made with reference to histology; 2, diceCT slices annotated based on epithelial thickness without reference to histology | | | | | | |
