# Supplementary material for: A comparison of diceCT and histology for determination of nasal epithelial type
Source: PeerJ. 2021 Nov 3;9:e12261. doi: 10.7717/peerj.12261 (PMC8571959; doi:10.7717/peerj.12261)
Supplement: Supplemental Information 7 [file peerj-09-12261-s007.docx]

| Table S4: Comparison of olfactory epithelial perimeter in *Desmodus* using two methods | | | | | | |
| --- | --- | --- | --- | --- | --- | --- |
| Perimeter (mm) of OE on roof/septum | | | | Perimeter (mm) of OE on ET I | | |
| Matching levels | histo-annotated^1^ | Blind^2^ | difference | histo-annotated | Blind | difference |
| 1 | 3.03 | 2.349 | 0.681 | 0.391 |  | 0.391 |
| 2 | 4.61 | 3.27 | 1.34 | 0.734 |  | 0.734 |
| 3 | 4.632 | 3.394 | 1.238 | 1.148 | 1.292 | -0.144 |
| 4 | 4.741 | 3.338 | 1.403 | 1.357 | 1.345 | 0.012 |
| 5 | 3.845 | 3.595 | 0.25 | 1.724 | 1.467 | 0.257 |
| 6 | 3.994 | 3.686 | 0.308 | 1.605 | 1.98 | -0.375 |
| 7 | 4.023 | 3.522 | 0.501 | 2.069 | 1.796 | 0.273 |
| 8 | 4.03 | 3.869 | 0.161 | 2.325 | 1.841 | 0.484 |
| 9 | 3.91 | 4.006 | -0.096 | 2.398 | 1.968 | 0.43 |
| 10 | 4.357 | 3.703 | 0.654 | 2.604 | 1.886 | 0.718 |
| 11 | 4.331 | 3.73 | 0.601 | 2.822 | 2.158 | 0.664 |
| 12 | 4.43 | 3.862 | 0.568 | 3.277 | 2.516 | 0.761 |
| 13 | 4.837 | 3.132 | 1.705 | 3.272 | 2.231 | 1.041 |
| 14 | 2.694 | 2.129 | 0.565 | 3.288 | 2.367 | 0.921 |
| 15 | 2.792 | 2.328 | 0.464 | 3.323 | 2.582 | 0.741 |
| 16 | 2.839 | 2.18 | 0.659 | 3.282 | 2.806 | 0.476 |
| 17 | 2.649 | 2.025 | 0.624 | 3.119 | 2.717 | 0.402 |
| 18 | 2.624 | 2.114 | 0.51 | 3.182 | 2.542 | 0.64 |
| 19 | 2.48 | 2.415 | 0.065 |  |  |  |
| 20 | 2.546 | 2.301 | 0.245 |  |  |  |
|  |  | average | 0.622 |  |  | 0.468 |
| 1, Based on annotations made with reference to histology; 2, diceCT slices annotated based on epithelial thickness without reference to histology | | | | | | |
