# Supplementary material for: A comparison of diceCT and histology for determination of nasal epithelial type
Source: PeerJ. 2021 Nov 3;9:e12261. doi: 10.7717/peerj.12261 (PMC8571959; doi:10.7717/peerj.12261)
Supplement: Supplemental Information 8 [file peerj-09-12261-s008.docx]

| Table S5: Comparison of olfactory mucosa^1^ perimeter in *Cynopterus* using two methods | | | | | | |
| --- | --- | --- | --- | --- | --- | --- |
| Perimeter (mm) of olfactory mucosa on roof/septum | | | | Perimeter (mm) of olfactory mucosa on the first ethmoturbinal | | |
| Matching slice | histo-annotated^2^ | Blindly marked slice^3^ | difference | histo-annotated | Blindly marked slice | difference |
| 1 | 3.7 | 3.431 | 0.269 | 2.605 | 2.424 | 0.181 |
| 2 | 3.604 | 3.394 | 0.21 | 2.504 | 2.349 | 0.155 |
| 3 | 3.409 | 3.419 | -0.01 | 2.36 | 2.287 | 0.073 |
| 4 | 3.49 | 3.31 | 0.18 | 2.313 | 2.262 | 0.051 |
| 5 | 3.471 | 3.352 | 0.119 | 2.49 | 2.26 | 0.23 |
| 6 | 3.328 | 3.172 | 0.156 | 2.081 | 2.116 | -0.035 |
| 7 | 3.384 | 3.116 | 0.268 | 2.22 | 2.194 | 0.026 |
| 8 | 3.288 | 3.122 | 0.166 | 2.184 | 2.2 | -0.016 |
| 9 | 2.906 | 2.992 | -0.086 | 2.264 | 2.077 | 0.187 |
| 10 | 2.825 | 2.879 | -0.054 | 2.223 | 2.133 | 0.09 |
| 11 | 2.979 | 2.803 | 0.176 | 2.129 | 2.112 | 0.017 |
| 12 | 2.513 | 2.609 | -0.096 | 2.135 | 2.014 | 0.121 |
| 13 | 2.872 | 2.549 | 0.323 | 2.215 | 2.035 | 0.18 |
| 14 | 2.846 | 2.488 | 0.358 | 2.004 | 2.074 | -0.07 |
| 15 | 2.505 | 2.436 | 0.069 | 1.801 | 1.971 | -0.17 |
| 16 | 2.687 | 2.411 | 0.276 | 1.955 | 1.954 | 0.001 |
| 17 | 2.641 | 2.345 | 0.296 | 1.859 | 1.873 | -0.014 |
| 18 | 2.655 | 2.201 | 0.454 | 1.985 | 1.833 | 0.152 |
| 19 | 2.252 | 2.027 | 0.225 | 2.017 | 1.896 | 0.121 |
| 20 | 2.273 | 2.03 | 0.243 | 2.036 | 1.906 | 0.13 |
| 21 | 2.192 | 1.954 | 0.238 | 2.102 | 1.871 | 0.231 |
| 22 | 1.985 | 1.826 | 0.159 | 1.931 | 1.792 | 0.139 |
| 23 | 2.125 | 1.745 | 0.38 | 1.766 | 1.782 | -0.016 |
| 24 | 2.144 | 1.527 | 0.617 | 1.671 | 1.735 | -0.064 |
| 25 | 1.677 | 1.517 | 0.16 | 1.607 | 1.763 | -0.156 |
| 26 | 1.513 | 1.319 | 0.194 | 1.512 | 1.748 | -0.236 |
| 27 | 1.21 | 1.347 | -0.137 | 1.497 | 1.423 | 0.074 |
| 28 | 1.018 | 1.21 | -0.192 | 1.434 | 1.502 | -0.068 |
| 29 | 0.93 |  | 0.93 | 1.015 | 1.343 | -0.328 |
| 30 | 0.879 |  | 0.879 | 1.382 | 1.086 | 0.296 |
| 31 | 0.518 |  | 0.518 | 1.053 | 1.02 | 0.033 |
| 32 | 0.395 |  | 0.395 | 1.243 | 0.944 | 0.299 |
| 33 |  |  |  | 1.02 | 0.862 | 0.158 |
| 34 |  |  |  | 0.887 | 0.604 | 0.283 |
| 35 |  |  |  | 0.422 | 0.444 | -0.022 |
| 36 |  |  |  | 0.32 | 0.375 | -0.055 |
| 37 |  |  |  |  | 0.438 | -0.438 |
|  |  |  |  |  |  |  |
| Average difference | | | 0.240 |  |  | 0.042 |
| 1, thick epithelium with immediately deep, opaque lamina propria are criteria; 2, Based on annotations made with reference to histology; 3, diceCT slices annotated based on epithelial thickness without reference to histology | | | | | | |
