# Supplementary material for: A comparison of diceCT and histology for determination of nasal epithelial type
Source: PeerJ. 2021 Nov 3;9:e12261. doi: 10.7717/peerj.12261 (PMC8571959; doi:10.7717/peerj.12261)
Supplement: Supplemental Information 9 [file peerj-09-12261-s009.docx]

| Table S6: Comparison of olfactory mucosa^1^ perimeter in *Desmodus* using two methods | | | | | | |
| --- | --- | --- | --- | --- | --- | --- |
| Perimeter (mm) of olfactory mucosa on roof/septum | | | | Perimeter (mm) of olfactory mucosa on the first ethmoturbinal | | |
| Matching levels | histo-annotated^2^ | Blind^3^ | difference | histo-annotated | Blind | difference |
| 1 | 3.03 | 2.721 | 0.309 | 0.391 |  | 0.391 |
| 2 | 4.61 | 3.876 | 0.734 | 0.734 | 1.041 | -0.307 |
| 3 | 4.632 | 3.985 | 0.647 | 1.148 | 1.39 | -0.242 |
| 4 | 4.741 | 4.119 | 0.622 | 1.357 | 1.4 | -0.043 |
| 5 | 3.845 | 4.111 | -0.266 | 1.724 | 1.434 | 0.29 |
| 6 | 3.994 | 3.694 | 0.3 | 1.605 | 1.734 | -0.129 |
| 7 | 4.023 | 4.249 | -0.226 | 2.069 | 1.89 | 0.179 |
| 8 | 4.03 | 4.051 | -0.021 | 2.325 | 2.065 | 0.26 |
| 9 | 3.91 | 3.882 | 0.028 | 2.398 | 2.25 | 0.148 |
| 10 | 4.357 | 3.947 | 0.41 | 2.604 | 2.322 | 0.282 |
| 11 | 4.331 | 3.933 | 0.398 | 2.822 | 2.533 | 0.289 |
| 12 | 4.43 | 4.386 | 0.044 | 3.277 | 2.914 | 0.363 |
| 13 | 4.837 | 4.503 | 0.334 | 3.272 | 2.988 | 0.284 |
| 14 | 2.694 | 2.888 | -0.194 | 3.288 | 3.204 | 0.084 |
| 15 | 2.792 | 3.14 | -0.348 | 3.323 | 3.408 | -0.085 |
| 16 | 2.839 | 3.07 | -0.231 | 3.282 | 3.409 | -0.127 |
| 17 | 2.649 | 2.839 | -0.19 | 3.119 | 3.468 | -0.349 |
| 18 | 2.624 | 2.785 | -0.161 | 3.182 | 3.461 | -0.279 |
| 19 | 2.48 | 2.749 | -0.269 |  |  |  |
| 20 | 2.546 | 2.694 | -0.148 |  |  |  |
|  |  |  |  |  |  |  |
|  |  | average | 0.089 |  |  | 0.056 |
| 1, thick epithelium with immediately deep, opaque lamina propria are criteria; 2, Based on annotations made with reference to histology; 3, diceCT slices annotated based on epithelial thickness without reference to histology | | | | | | |
